# Functional *Allium fistulosum* Centromeres Comprise Arrays of a Long Satellite Repeat, Insertions of Retrotransposons and Chloroplast DNA

**DOI:** 10.3389/fpls.2020.562001

**Published:** 2020-10-23

**Authors:** Ilya Kirov, Sergey Odintsov, Murad Omarov, Sofya Gvaramiya, Pavel Merkulov, Maxim Dudnikov, Alexey Ermolaev, Katrijn Van Laere, Alexander Soloviev, Ludmila Khrustaleva

**Affiliations:** ^1^Laboratory of Marker-assisted and genomic selection of plants, All-Russia Research Institute of Agricultural Biotechnology, Moscow, Russia; ^2^Kurchatov Genomics Center of ARRIAB, All-Russia Research Institute of Agricultural Biotechnology, Moscow, Russia; ^3^Center of Molecular Biotechnology, Russian State Agrarian University-Moscow Timiryazev Agricultural Academy, Moscow, Russia; ^4^National Research University Higher School of Economics, Moscow, Russia; ^5^Flanders Research Institute for Agriculture, Fisheries and Food (ILVO), Plant Sciences Unit, Melle, Belgium; ^6^Plant Cell Engineering Laboratory, All-Russia Research Institute of Agricultural Biotechnology, Moscow, Russia

**Keywords:** chromosomes, tandem repeats, fish, retrotransposons, allium, centromere, chloroplast insertions

## Abstract

The centromere is a unique part of the chromosome combining a conserved function with an extreme variability in its DNA sequence. Most of our knowledge about the functional centromere organization is obtained from species with small and medium genome/chromosome sizes while the progress in plants with big genomes and large chromosomes is lagging behind. Here, we studied the genomic organization of the functional centromere in *Allium fistulosum* and *A. cepa*, both species with a large genome (13 Gb and 16 Gb/1C, 2*n* = 2*x* = 16) and large-sized chromosomes. Using low-depth DNA sequencing for these two species and previously obtained CENH3 immunoprecipitation data we identified two long (1.2 Kb) and high-copy repeats, AfCen1K and AcCen1K. FISH experiments showed that AfCen1K is located in all centromeres of *A. fistulosum* chromosomes while no AcCen1K FISH signals were identified on *A. cepa* chromosomes. Our molecular cytogenetic and bioinformatics survey demonstrated that these repeats are partially similar but differ in chromosomal location, sequence structure and genomic organization. In addition, we could conclude that the repeats are transcribed and their RNAs are not polyadenylated. We also observed that these repeats are associated with insertions of retrotransposons and plastidic DNA and the landscape of *A. cepa* and *A. fistulosum* centromeric regions possess insertions of plastidic DNA. Finally, we carried out detailed comparative satellitome analysis of *A. cepa* and *A. fistulosum* genomes and identified a new chromosome- and *A. cepa*-specific tandem repeat, TR2CL137, located in the centromeric region. Our results shed light on the *Allium* centromere organization and provide unique data for future application in *Allium* genome annotation.

## Introduction

The centromere plays a key role in proper chromosome segregation during cell division. In spite of its conservative function, centromeric DNA exhibits large variability among eukaryotic genomes (Jiang et al., 2003; Talbert and Henikoff, 2020). The conservative function of centromeres and, in contrast, the diversity of their structures is one of the enigmas of modern biology. Although many advanced sequencing and scaffolding technologies are available, the full-length assembly of highly repetitive centromere sequences is still a challenging task. Centromeres with a short array of repetitive DNA or with frequent insertions of mobile elements creating a unique genomic pattern, are the most suitable for assembly. Such centromeres were found in several plant species and were well assembled (Feng et al., 2002; Yan et al., 2008; Wolfgruber et al., 2009; Gent et al., 2017). Although long-read sequencing technologies and modern algorithms facilitated centromere assembly (Bzikadze and Pevzner, 2020), in most of the assembled genomes the centromeric sequences are underrepresented because they mostly are part of the unanchored set of contigs (Saint-Oyant et al., 2018; Su et al., 2019). Therefore, our knowledge about the centromere sequence organization is rudimentary.

In most eukaryotes, the position of the centromere is epigenetically determined by the specific variant of histone H3, CENH3, which is a hallmark of the functional centromere. CENH3 containing nucleosomes are involved in kinetochore formation (Blower et al., 2002). However, also CENH3-independent kinetochore assembly pathways have been reported in insects (Drinnenberg et al., 2014; Mon et al., 2017) and in plants (Oliveira et al., 2020). Using antibodies against CENH3, DNA sequences, comprising the functional centromere, were isolated for many plant species (Jiang et al., 2003; Yan et al., 2008; Gong et al., 2012; Melters et al., 2013; Zhang et al., 2013; Robledillo et al., 2018). These studies showed that centromeres may consist of repetitive DNA and/or unique DNA sequences with the latter ones being signs of neo-centromeres. Centromeres may also comprise functional genes and multiple insertions of plastidic (NUPTs) and mitochondrial (NUMTs) genomes (Sullivan and Karpen, 2004; Yan et al., 2008; Michalovova et al., 2013). However, whether these elements are involved in centromere function or are located in CENH3-free loci is not well established. Overall, CENH3-associated sequences of most of the plant species studied to date belong to two types of repetitive sequences, namely, centromere specific retrotransposons (CRs) and centromeric tandem repeats (CTRs), although other repeat families and unique sequences can also be involved (Jiang et al., 2003; Sharma and Presting, 2008; Gong et al., 2012; Su et al., 2016). CRs belong to chromoviruses (Chromoviridae), a specific lineage of Ty3/Gypsy retrotransposons, which possess an integrase chromodomain that targets the insertion in centromeric chromatin (Neumann et al., 2011). CRs can be associated with CENH3 histones as a unique member of the functional centromere as it was shown for *Brassica nigra* (Wang et al., 2019) or can be intermixed with CENH3-associated CTRs (Zhong et al., 2002; Kowar et al., 2016). CTRs were characterized in many plant species and their rapid divergence between species or even between chromosomes of a single set was demonstrated (Henikoff et al., 2001; Gong et al., 2012; Melters et al., 2013; Zhang et al., 2013; Robledillo et al., 2020). CTRs are organized into a long array of repeats with thousands of copies in the genome. Depending on the centromere type, CTR arrays may contain from thousands to million base pairs, but only a certain fraction of the repeats are capable to associate with CENH3 (Dawe, 2003; Houben et al., 2007; Yan et al., 2008). The monomer size seems to be an important characteristic of CTRs for CENH3 nucleosome stability (Hasson et al., 2013; Zhang et al., 2013; Yang et al., 2018) and usually is between 100 and 200 bp (Melters et al., 2013). However, large-scale analysis of CTRs across different plant and animal species showed a broad diversity both in CTR monomer size and in GC content (Melters et al., 2013; Robledillo et al., 2020). The maximum monomer length of CTRs was found in *Bos taurus taurus* (1,419 bp; (Melters et al., 2013) for animals and in *Fabeae* (2,033–2,979 bp; Robledillo et al., 2018, 2020) and *Solanacea* (5,390 bp; Gong et al., 2012) for plants. *Vicia faba* is a species with a large monomer length, a large genome size (∼13 Gbp; Bennett and Smith, 1976) and large chromosomes. In contrast, in potato, a species with small chromosomes and relatively small genome size (844 Mb, Bennett and Leitch, 1997), the monomer size ranges from 979 to 5,390 bp. The question arises whether the size of the centromeric monomer is correlated with the genome size and chromosome size, or whether this is a random result of the evolution of individual taxa. The numbers of characterized CTRs in plant species is still insufficient to answer this question.

Centromere sequencing demonstrated the presence of transcribed genes providing evidence that centromeric chromatin can be transcribed (Yan et al., 2008; Gent et al., 2017). Analysis of epigenetic marks enriched in transcriptionally active regions (H3K4me2, H3K36me2) showed that they are also present in the centromeric region (Sullivan and Karpen, 2004). There is also ample evidence that CTRs and CRs of eukaryotes are transcribed (Dawe, 2003; Topp et al., 2004; Neumann et al., 2011; Hall et al., 2012; Rosic and Erhardt, 2016; Talbert and Henikoff, 2018). Moreover, it was shown that transcription is essential for facilitating CENH3 loading and centromere initiation (Topp et al., 2004; Talbert and Henikoff, 2018; Ideue and Tani, 2020). The generated centromere transcripts of different organisms range in length (from tens to hundreds bases), post-transcriptional modifications (polyadenylation and capping) and their location (reviewed by Ideue and Tani, 2020). Centromere transcripts have been identified in maize (Topp et al., 2004) and *Arabidopsis* (May et al., 2005).

*Allium* species are important vegetables distributed world-wide (Brewster, 2008). In spite of the fact, that some *Allium* species are well known model plants in cytology there is only limited information available about their functional centromere sequences (Nagaki et al., 2012). Large *Allium* chromosomes are useful to check possible correlations between chromosome size and CTR monomer length. Pioneering work on ChIP isolation of the centromere repeat in *Allium fistulosum* has been performed by Nagaki et al. (2012), showing that several CENH3 associated repetitive sequences in the *A. fistulosum* genome (Afi sequences) are located in the centromeres of all chromosomes. However, the organization and origin of these CENH3 associated sequences remain unclear. Based on the known Afi sequences, here we conducted a molecular cytogenetic and bioinformatic study of the CENH3-associated centromeres in *A. fistulosum* and *A. cepa*. Our results showed that the centromere of *A. cepa* and *A. fistulosum* possess include long (∼1.25 Kb) tandem repeats (AcCen1K and AfCen1K) with some structural differences between them. We showed that the centromeres of the two *Allium* species contain repeat sequences with partial similarity but differ in chromosomal location, sequence structure and genomic organization. We demonstrated that these repeats are transcribed and that their transcripts are not polyadenylated. We also found that the centromere regions of these species possess insertions of retrotransposons and organelle DNA.

## Materials and Methods

### Plant Material, Chromosomes Preparation and DNA Isolation

*Allium fistulosum* L. “Russkiy Zimniy” (2*n* = 2*x* = 16) and *A. cepa* L. “Haltsedon” (2*n* = 2*x* = 16) seeds were purchased from “Gavrish” seed company (Moscow, Russian Federation). Genomic DNA was isolated from 5-day-old seedlings and young leaves of *A. cepa* and *A. fistulosum* according to the previously described method of (Rogers and Bendich, 1985). Mitotic chromosomes were prepared according to the “SteamDrop” protocol (Kirov et al., 2014).

### PCR Amplification of the Repeats and Cloning

To identify the functional centromere unit of *Allium fistulosum*, we explored CENH3-associated sequences previously determined for this species (NCBI accession numbers: AB735741–AB735747; Nagaki et al., 2012). To check whether they have similarity to CRM or other retrotransposon sequences we performed a BLASTX search to known domains of retrotransposons (“core” database) from the GyDb database^1^ (Llorens et al., 2010) using build-in BLASTX tool with default parameters. Primers were designed by Primer 3.0 plus software^2^. Primers used for the PCR amplification of Afi11 sequence revealed by (Nagaki et al., 2012) InDel and new tandem repeat of *A. cepa* (TR2CL137) are listed in Table 1.

**TABLE 1:** Primers used in this study and the expected PCR product size.

Tandem repeat	Primers 5′–3′	Estimated length of PCR product, bp
Afi11 (GenBank: AB735740)	F: AAAGGTTCATGCCTGCTTTC R: TTTTACGGCATGCGATACCT	111
TR2CL137/230 (276bp)	F: CTGCATATTTTCGCATAATCTTTCACG R: ATGCAAACTAACGTGAAAATGTGA	150
InDel primers	F: GACGAAACTGGCGCCATCG R: TCTTGTATGTTATCACCGTTTAGTG	∼430 bp (*A. fistulosum* DNA)/1,100 bp (*A. cepa* DNA)

The following PCR conditions were used: 94°C – 1 min, 30 cycles: 94°C – 1 min; 58°C – 1 min; 72°C – 1 min; final elongation: 72°C – 5 min. The PCR mixture consisted of 0.5 μL of 2.5 mM mixture of dinucleotides (dNTPs), 10× PCR buffer (Evrogen), 2.5 mM MgCl_2_ and 0.5 μL of 10 μM solution of forward and reverse primers and 0.25 μL of Taq polymerase (Evrogen).

The PCR product with Afi11 primers and *A. fistulosum* DNA was cloned into the pCR2.1-TOPO vector using the manufacturer’s protocol (Invitrogene). The PCR products with AfCen1K primers and *A. cepa* DNA was cloned into pAL-T (Evrogen, Moscow) vector following manufacturer’s instructions. The sequences of the repeats are available at NCBI database under following accessions: MT374062 (AfCen1K) and MT374061 (AcCen1K).

### *De novo* Repeat Identification Using Illumina Reads and Sequence Analysis

Whole genome sequencing for *A. cepa* and *A. fistulosum* was performed by BGI on Illumina HiSeq4000. Insert size for library preparation was <500 bp. About 40 million high-quality 150 bp paired-end reads were filtered based on quality and adapters were removed using Trimmomatic v0.39 (Bolger et al., 2014) with settings: SLIDINGWINDOW:4:15 HEADCROP:15 MINLEN:150. Final read quality was estimated in FastQC (Andrews, 2010). All reads have 150 bp length after filtering and trimming. Then, 3% of the read pairs were randomly selected by a custom made golang program^3^ and used as input for RepeatExplorer2 (Novak et al., 2013) clustering analysis (∼0.02× genome coverage). The reads were combined into single fasta file and RepeatExplorer + TAREAN (included into RepeatExplorer2 software) were run in comparative mode with the following setting: -p -c 150 -C -r 400000000 -P 2. This analysis was also carried out separately for each species (settings: -p -c 150 -r 400000000) allowing to increase the number of reads and coverage. In total 1.206.269 read pairs were used in the clustering analysis. However, for *A. fistulosum* less than 50% of the reads were taken by RepeatExplorer2 software itself because a significant portion of its genome is occupied by a single tandem subtelomeric repeat resulting in higher demand in RAM. Therefore, to not cause a bias in repeat abundance estimation, the analysis in *A. cepa* in RepeatExplorer2 was repeated with an equal number of reads as for *A. fistulosum* (i.e., 641236). This analysis showed comparable results as for the first *A. cepa* repeat analysis. For the repeat family annotation, the automatic classification procedure provided by RepeatExplorer2 was exploited. Fisher’s exact test was performed Rstudio^4^ Version 1.2.1335 with R version 3.6.0.

To identify *A. cepa* RepeatExplorer cluster corresponding to AfCen1K BLASTn search (default settings and –outfmt 6) was performed with AfCen1K sequence as a query. Contigs assembled by RepeatExplorer2 after run with *A. cepa* reads were used as database. The resulted table was filtered by >90% identity and *E*-value <1e−5.

Transcription start sites (TSS) and *Cis*-regulatory elements were predicted using online tools, TSSPlant (Shahmuradov et al., 2017) and plantCARE (“Search for CARE” function) with default parameters, respectively.

### Paired-Read Data Analysis

To verify the tandem organization of AcCen1K and AfCen1K repeats in the genomes of *A. cepa* and *A. fistulosum*, paired reads were separately mapped to the reference sequences of the tandem repeats by bowtie2 version 2.3.4.3 (Langmead and Salzberg, 2012) with the following settings: –no-unal -k 10 –local. Obtained sam files for “left” and “right” read files were parsed using a custom made python script^5^ to extract the location of concordantly and unconcordantly mapped reads. The obtained table file was then used for visualization in shinyCircos (Yu et al., 2018). For identification of mobile elements and chloroplast DNA insertions, the obtained sam files were parsed by a custom made python script to find out the read pairs for which one read was mapped to the reference centromere sequence while the second read of the pair was not mapped to the reference. We used these reads in a blastn v. 2.8.1+ (default settings with -outfmt 6) search for similarity to the *Allium* chloroplast DNA (GenBank accession: KM088013.1), mitochondrial DNA (GenBank accession: NC_030100.1) and repeat sequence library that was constructed and annotated by RepeatExplorer2. The hits with identity >80% and *e*-value <1e − 05 were selected for further analysis. The filtered blast results were e then analyzed in Rstudio Version 1.2.1335 (see text footnote 4) with R version 3.6.0 using relevant packages including ggplot2 (Wickham, 2016) and data.table (Dowle et al., 2020).

### RNAseq Analysis

To estimate the transcription of AcCen1K and AfCen1K centromeric repeats by RNAseq publicly available data was used (Table 2).

**TABLE 2:** Publicly available RNAseq data used in this study.

SRA accession	Read number	Species	References
SRR1312066	74147085	*A. cepa*	Kim et al. (2015b)
SRR1312067	57281625	*A. cepa*	Kim et al. (2015b)
DRR006306	13005682	*A. fistulosum*	Tsukazaki et al. (2015)
DRR006298	1348977	*A. fistulosum*	Tsukazaki et al. (2015)
DRR006305	13872409	*A. fistulosum*	Tsukazaki et al. (2015)
DRR006304	13445096	*A. fistulosum*	Tsukazaki et al. (2015)
DRR006303	12926682	*A. fistulosum*	Tsukazaki et al. (2015)
DRR006302	13155452	*A. fistulosum*	Tsukazaki et al. (2015)
DRR006301	13874202	*A. fistulosum*	Tsukazaki et al. (2015)
DRR006300	13835914	*A. fistulosum*	Tsukazaki et al. (2015)
DRR006299	13486735	*A. fistulosum*	Tsukazaki et al. (2015)

The reads were filtered based on quality and reads were trimmed to remove adapters using Trimmomatic v0.39 (Bolger et al., 2014) with settings: SLIDINGWINDOW:4:15 HEADCROP:12. Final read quality was estimated in FastQC (Andrews, 2010). A database for mapping was prepared by combining the merged centromeric monomer and the reference transcriptome sequences for each species. Reference transcriptomes for *A. cepa* (cultilvars H6 and SP3B) and *A. fistulosum* were obtained from http://onion.snu.ac.kr and NCBI (TSA accession numbers: FX553726–FX608587 and FX657476–FX657516), respectively. To determine transcripts of actin gene in *A. cepa* and *A. fistulosum* we used Blast (TBLASTN 2.9.0+) with default options with actin sequence of *A. cepa* from Uniprot (D3YLT7) as query and reference transcriptomes for each species as databases. Top hits were chosen as contigs derived from actin gene. RNA-seq data from the same publications as reference transcriptomes *A. cepa* (Kim et al., 2015b) and *A. fistulosum* (Tsukazaki et al., 2015) were used to assess the transcription of AfCen1K and AcCen1K tandem repeats. Mapping of the RNAseq reads to the database sequences was carried out by Hisat2 (Kim et al., 2015a) using option “-k 200” to allow multihit mapping. Then number of reads mapped to actin gene and tandem repeats for both species were counted with HTseq (Anders et al., 2015) (settings: htseq-count –stranded = no) and TPM (transcripts per million) values were calculated.

BLASTn search (blastn v. 2.8.1+) of similarity of RNAseq reads against the AcCen1K and AfCen1K repeats was carried out with default settings and -outfmt 6. Hits were filtered using 90% similarity cutoff and *E*-value <1e − 05.

### RNA Isolation and RT-PCR

RNA was isolated from 5-days old seedlings of *A. cepa* and *A. fistulosum* using ExtractRNA kit (Evrogen, Moscow) following the manufacturer’s instructions. The RNA quality and quantity were estimated by gel electrophoresis using an 1.2% agarose gel with ethidium bromide staining. The RNA concentration was quantified using a Nanodrop (Nanodrop Technologies). Primers used for RT-PCR analysis of centromere repeat expression are listed in Table 1. The *Tubulin* gene was used as a reference gene with following primer pair: AcTub/F: CGTGACACCACAATTATCGCAAACACA; AcTub/R: TGTG AAATCAACGGTTTGCGACATTCC (Kim et al., 2004). For estimation of the expression in poly-A fraction, a PCR reaction was carried out with cDNA synthesized using the MMLV RT Kit with oligo-dT primers (Evrogen, Russia). RT-PCR with total RNA was performed using BioMaster RT-PCR-Color (2×) reagent kit (Biolabmix, Novosibirsk). PCR reactions on MQ and DNAse-treated RNA was done as negative controls. The PCR products were visualized by gel electrophoresis on an 1.2% agarose gel with ethidium bromide staining.

### Probe Labeling, Fluorescent *in situ* Hybridization (FISH) and Microscopy

Probes for fluorescence *in situ* hybridization (FISH) were made by PCR labeling (FISH with TR2CL137 repeat) using Biotin-16-dUTP (Roche, Mannheim, Germany) and Taq Polimerase (Evrogen) or by Biotin-Nick Translation Mix (the PCR products from long-range PCR amplification with AfCen1K primers and *A. fistulosum* genomic DNA, AcCen1K plasmids and chloroplast DNA BAC clone) according to the manufacturer’s protocol (Roche, Mannheim, Germany). BAC clone contacting chloroplast DNA insertion of barley was kindly provided by thank Dr. Andreas Houben. FISH was performed as described in (Kirov et al., 2017b). The Biotin-labeled probe was detected with streptavidin-Cy3 (Sigma–Aldrich, St. Louis, MO, United States). Chromosomes were counterstained in 5 μg/ml DAPI in Vectashield anti-fade (Vector Laboratories, United States). Slides were checked using a Zeiss Axio Imager microscope M1 (Carl Zeiss MicroImaging, Jena, Germany) and 5–10 metaphases per slide were used for the analysis. At least three biological replicates (independent FISH experiments) were involved for each probe. Images were captured using an Axio Cam MRm digital camera. Image processing was performed using AxioVision version 4.6 software (Carl Zeiss MicroImaging, Jena, Germany). The captured images of the chromosomes were measured using DRAWID^6^ software version 0.26 (Kirov et al., 2017a).

## Results

### Sequencing of Full-Length Centromeric Tandem Repeat of *A. fistulosum*

**FIGURE 1:**
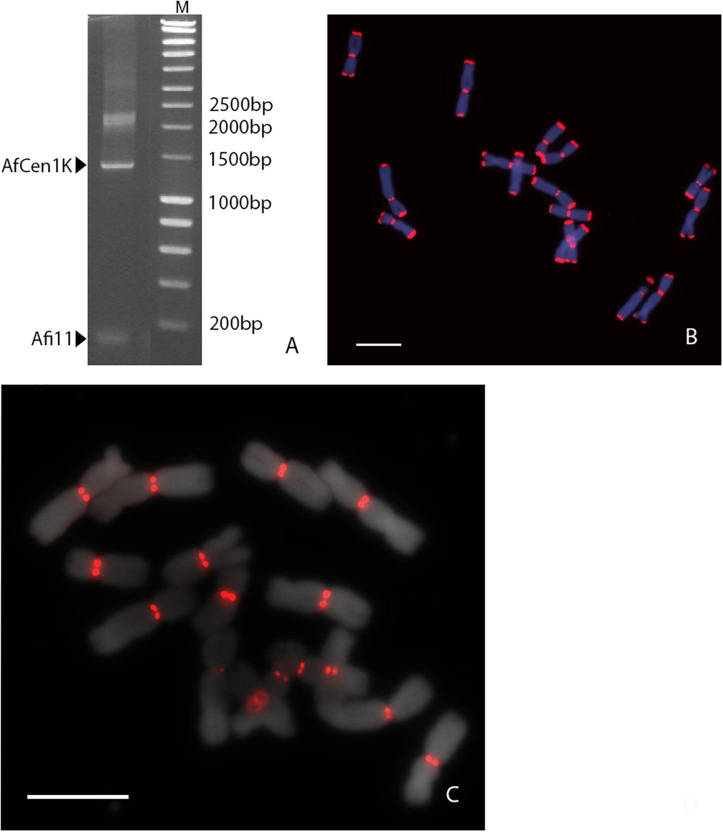
Identification of full-length *A. fistulosum* centromere repeat sequence. **(A)**. Long-range PCR amplification with Afi11 (CENH3-associated fragment) primers on genomic DNA of *A. fistulosum*. PCR products corresponding to original Afi11 fragment (158 bp) and full-length centromeric repeat (AfCen1K, <1,249 bp) are indicated (M – GeneRuler^TM^ 1 Kb DNA Ladder). FISH on *A. fistulosum* mitotic metaphase chromosomes with the labeled PCR product from long-range PCR amplification with Afi11 primers **(B)** and with an individual plasmid carrying the full-length AfCen1K (bar = 10 μm) **(C)**.

Earlier Nagaki et al. (2012) reported *A. fistulosum* centromere sequences (Afi sequences) which showed no sequence homology to those in the NCBI GenBank database. Our search in the NCBI nucleotide database by BLASTn confirmed the absence of sequences similar to the Afi centromere sequences in other organisms. We then assessed whether the *Allium* centromere comprises tandem repeat sequences. For this, long-range PCR amplification was done with specific primers designed for one of the Afi (Afi11) sequences on *A. fistulosum* genomic DNA. This resulted in amplification one small fragment with the expected length (111 bp) corresponding to Afi11 and a ladder-like longer PCR fragment of about 1 Kb (Figure 1A). To validate that the long PCR fragments are centromeric sequences we carried out FISH with the labeled total PCR product. Interestingly, FISH showed clear signals both in the centromeric and the subtelomeric regions (Figure 1B). The signals in the centromeric region were much stronger than ones obtained with labeled Afi11 PCR product alone in distinct experiment (data not shown). The PCR product was cloned into a plasmid vector and sequenced. FISH with an individual clones carrying kilobase-size insertions resulted in signals only located on the centromeres of all *A. fistulosum* chromosomes (Figure 1C). No signals were observed in the subtelomeric regions suggesting that the initial PCR product contained traces of high-copy subtelomeric repeats or derivatives (e.g., microsatellites; Fesenko et al., 2002) which probably are co-located with the centromere repeat in the *A. fistulosum* genome. Sequence analysis of five clones revealed sequences with a length between 1,239 and 1,259 bp and 92–97% similarity (Supplementary File S1). The GC content of the sequences was 32%. This repeat was named “AfCen1K” (GenBank accession number: MT374062). BLASTn search of AfCen1K similarity to known sequences deposited at NCBI did not reveal any significant hits except for Afi sequences that aligned along the whole length of the AfCen1K sequence suggesting that all parts of AfCen1K are able to interact with CENH3 histone.

### *A. fistulosum* (AfCen1K) and *A. cepa* (AcCen1K) Repeats Differ in Structure and Genome Organization

**FIGURE 2:**
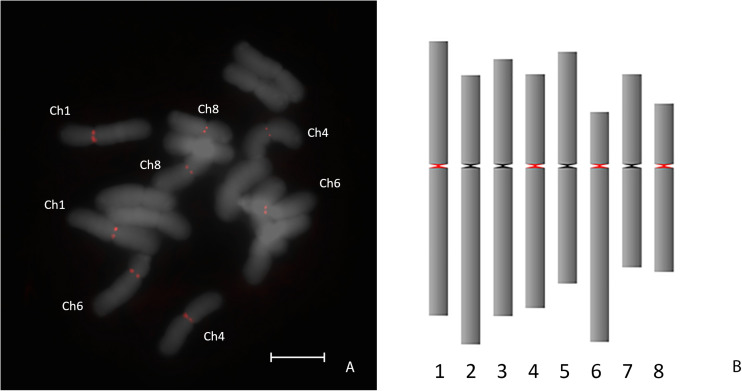
FISH mapping of AfCen1K on *A. cepa* chromosomes. The chromosomes possessing FISH signals are assigned **(A)**. Ideogram with marked (red) positions of AfCen1K FISH signals (bar = 10 μm) **(B)**.

To determine if AfCen1K is also presents in the genome of bulb onion (*A. cepa*), FISH with the labeled AfCen1K clone was performed on *A. cepa* and revealed signals located at the centromeres of four *A. cepa* chromosome pairs (Figure 2A): chromosome 1, 4, 6, and 8 (Figure 2B). Using the AfCen1K sequence we searched for similarity in the *A. cepa* repeatome and satellitome. For this, we exploited the graph-based clustering of Illumina reads followed by cluster annotation and tandem repeat search in RepeatExplorer2 (Novak et al., 2013) and TAREAN tools (Novák et al., 2017). In total, 40 million high-quality paired-end reads were obtained and 3% of the reads were randomly selected and used for the analysis (∼0.02× genome coverage). Read clustering followed by manual and automatic annotations showed that 69% *A. cepa* (AC) reads were placed in clusters. With this coverage, only middle- and high-copy repeats are detected. So the actual portion of all repetitive sequences in *Allium* genomes is above 70%. We then performed a search for clusters corresponding to AfCen1K centromeric sequences and found one cluster (CL85, Supplementary File S2) with significant similarity to AfCen1K. Based on the number of reads (1,017 reads) in this cluster the genome portion occupied by AfCen1K was calculated to be up to 0.079% corresponding to ∼7,200 copies/1C.

**FIGURE 3:**
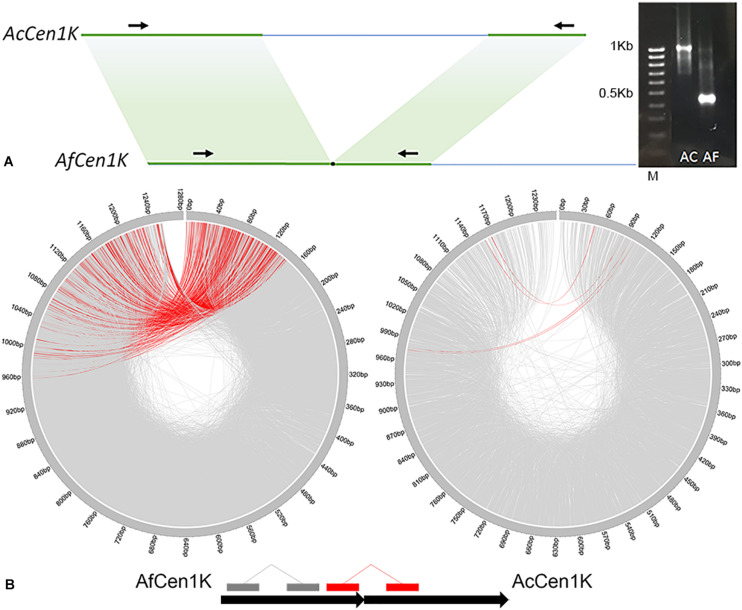
Comparative analysis of AfCen1K and AcCen1K monomer structure. **(A)** Scheme showing the similar regions between AfCen1K and AcCen1K sequences (green area); Primer positions (black arrows) and gel electrophoresis of the PCR products obtained with these primers for *A. cepa* (AC) and *A. fistulosum* (AF) genomic DNA are shown (M – 100 bp DNA Ladder (Syntol)). **(B)** Circos plot demonstrating the read pairs mapping concordantly (gray lines) and discordantly (red lines) to the reference sequence. The schematic positions of the concordantly and discordantly mapped reads on the two tandemly repeated Af(Ac)Cen1K sequences are illustrated below.

To check whether this data can be used to assemble the *A. cepa* AfCen1K-like centromeric repeat we performed *de novo* assembly of AfCen1K. For this RepeatExplorer2 run was carried out with *A. fistulosum* reads followed by AfCen1K cluster identification and sequence assembly. Sequence comparison of the AfCen1K assembled contig (1,230 bp) from the corresponding AF cluster (CL85) with the reference AfCen1K sequence showed high similarity (94%) and query coverage (100%). This comparison suggested that assembly of the *Allium* centromere monomer from short reads using the current number of reads provides correct results for *A. fistulosum*. We then used the same strategy for *A. cepa* and found one cluster (CL157, Supplementary File S2) with reads that are similar to AfCen1K. Using these reads we assembled the 1,255 bp sequence (hereinafter termed AcCen1K, GenBank accession number: MT374061) of the *A. cepa* centromere monomer. Based on the number of reads (528 reads) in the cluster the genome portion occupied by AcCen1K was calculated to be up to 0.021% of the *A. cepa* genome corresponding to ∼2,600 copies/1C. Comparison of AfCen1K with AcCen1K sequence showed a high partial similarity (90–94%) of a near 600 bp fragment (50% length of initial sequences) while other parts of the sequences have no similarity (Figure 3A). In addition, an InDel polymorphism (632 bp) was detected between AcCen1K and AfCen1K sequence. To prove these results, we designed primers on the conserved parts of the two sequences flanking the polymorphic InDel region (Figure 3A). As it was expected, the PCR resulted in the amplification of 1,100 and 430 bp fragments from *A. cepa* and *A. fistulosum* genomic DNAs, respectively, proving the presence of an InDel which distinguishes the centromeric repeat sequences of the two *Allium* species.

AcCen1K PCR product was cloned and the sequence was verified by Sanger sequencing. To determine the physical position of this AcCen1K in *A. cepa* genome we performed FISH with *A. cepa* and *A. fistulosum* chromosomes. Surprisingly, no FISH signals from labeled AcCen1K were detected on *A. cepa* chromosomes (Supplementary Figure 2) although on *A. fistulosum* chromosomes weak signals with this probe were obtained (Supplementary Figure 3). These results suggested that AcCen1K repeat does not form any long-size arrays in *A. cepa* genome that exceed FISH sensitivity cutoff (>10 Kb).

To further study the genomic organization of AcCen1K in *A. cepa* genome, we exploited the genomic paired reads of *A. cepa* and *A. fistulosum* and mapped them to the AcCen1K and AfCen1K sequences, respectively. In total, 7,585 and 1,735 read pairs were mapped to the AfCen1K and AcCen1K sequences, respectively. For *A. fistulosum*, 413 (5.4%) reads pairs mapped disconcordantly, one read of a pair mapped in the “head” and one read in the “tail” of the centromere sequences, suggesting tandem organization of AfCen1K in the *A. fistulosum* genome. In contrast, only 4 (0.2%) *A. cepa* read pairs mapped to the opposite positions on the target sequence, suggesting less frequent tandem organization for the AcCen1K compared to the AfCen1K centromeric repeat.

Thus, we showed that the centromeres of the two *Allium* species contain repeat sequences with partial similarity but differ in sequence structure and chromosome organization.

### *Allium* Centromere Contains Chloroplast Sequence and Mobile Element Insertions

**FIGURE 4:**
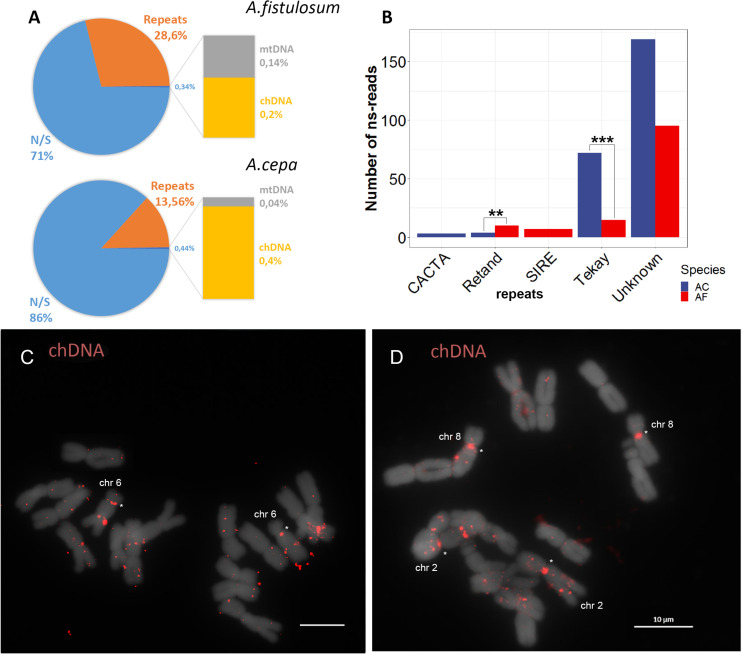
Ns-read analysis of *A. cepa* and *A. fistulosum* centromeres. **(A)** Pie chart showing the number of ns-reads similar to repetitive sequences (Repeats), chloroplast DNA (chDNA), mitochondrial DNA (mtDNA) and ns-reads without similarity (N/S). **(B)** Bar plot of number of ns-reads of the two species with similarity to different mobile element lineages; ** and *** marks <0.01 and <0.001 Fisher’s exact test *P*-values, respectively. **(C,D)** FISH with labeled BAC clone possessing chloroplast DNA on chromosomes of *A. cepa* **(C)** and *A. fistulosum* (bar = 10 μm) **(D)**.

To get a closer look to the other DNA sequences located on the *Allium* centromere, we performed analysis of read pairs where one read was mapped to the centromere repeats (AcCen1K or AfCen1K) while the other read was not similar to the identified centromeric sequences (ns-read). Totally, 2,314 and 2,373 ns-reads were collected for *A. fistulosum* and *A. cepa*, respectively. Three groups of sequences were used to identify similarity with ns-reads including chloroplast DNA (GenBank accession: KM088013.1), mitochondrial DNA (GenBank accession: NC_030100.1) and the repeat library constructed by RepeatExplorer2 (see above). A bulk portion of ns-reads (86% for *A. cepa* and 71% for *A. fistulosum*) did not have similarity to any of the target sequences. But 13% of *A. cepa* ns-reads and 28% of *A. fistulosum* ns-reads do have similarity to repetitive DNA (Figure 4A). Annotation of the high-copy repeats (occupying >0.05% of the genome) with similarity to ns-reads showed that they belong to either unknown repeats (since classification is not possible using a similarity based approach), Ty3/Gypsy (Tekay and Retand lineages), Ty1/Copia (SIRE lineage) or DNA transposons (CACTA family) (Figure 4B). ns-reads similar to SIRE mobile elements were found for *A. fistulosum* and to CACTA for *A. cepa*. Ns-reads with similarity to Tekay and Retand lineages of Ty3/Gypsy were found in datasets of both species but their numbers differ substantially with significant overrepresentation for both lineages in *A. cepa* compared to *A. fistulosum* (Fisher’s exact test *p*-values: 0.001 for Tekay and 0.008 for Retand) (Figure 4B).

In addition, 0.14% of *A. fistulosum* and 0.04% of *A. cepa* ns-reads were similar to mitochondrial DNA. Interestingly, 0.2 and 0.4% of *A. fistulosum* and *A. cepa* reads showed similarity to chloroplast DNA sequences suggesting possible insertion of chloroplast DNA into the nucleus. To find further evidence for this, we performed FISH experiments with labeled BAC clones containing an insertion of barley chloroplast DNA (provided by Dr. Andreas Houben). The results of this experiment demonstrated multiple insertion of chloroplast DNA into the nuclear genome in both species with some insertions occurring in centromeric regions (Figures 4C,D).

### A New Centromere Specific Tandem Repeat in *A. cepa*

**FIGURE 5:**
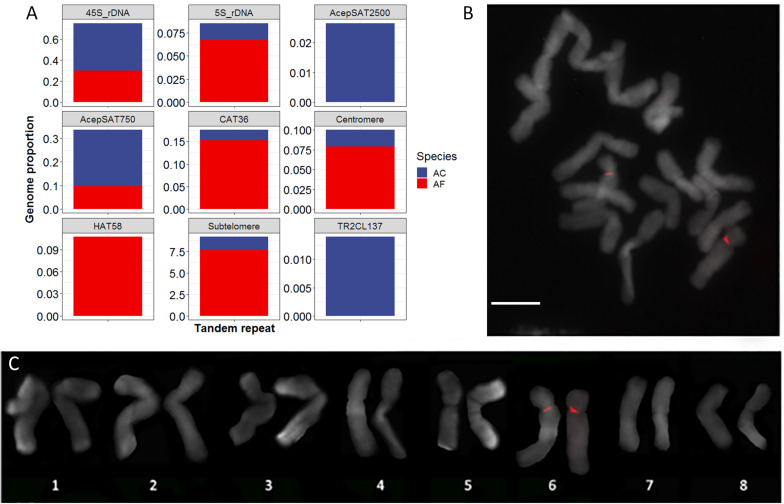
Tandem repeats of *A. cepa* and *A. fistulosum* genomes. **(A)** Genome proportion of known and new (TR2CL137) tandemly organized sequences in *A. cepa* (AC) and *A. fistulosum* (AF) genomes; **(B)** FISH with labeled TR2CL137 tandem repeat on *A. cepa* chromosomes (bar – 10 μm); **(C)** Karyotype of *A. cepa* chromosomes after FISH with labeled TR2CL137 repeat.

To find tandem repeats that are located in the centromere regions of *A. cepa* and *A. fistulosum*, we performed a comparative analysis of repetitive sequences of both species using RepeatExplorer2 and TAREAN tools. We then classified the repetitive sequences according to their sequence homology to known repeat families (e.g., mobile elements) and to genome organization features (tandem repeats). The biggest fraction of the repeatome belongs to the unknown repeat families (AC: 28%, AF: 22%). From the classifiable repeats, the highest portion belongs to Ty3/Gypsy retrotransposons, accounting for 8 and 9% in AC and AF genomes, respectively. We classified the mobile elements in the genomes more deeply and found four Ty3/Gypsy lineages, namely Tekay (chromovirus), TatV (non-chromovirus/OTA), CR (chromovirus) and Athila (non-chromovirus/OTA), and three lineages of Ty1/Copia (TAR, SIRE, and Tork) that are significantly represented in the two *Allium* genomes. We then annotated clusters corresponding to Tandem Repeat sequences. BLAST with known *A. cepa* and *A. fistulosum* tandem repeat sequences (Kirov et al., 2017b; Peška et al., 2019) and AfCen1K and AcCen1K were used to annotate Tandem Repeat clusters. Clusters of eight major *Allium* tandemly organized repeats including a subtelomeric repeat, 5S rDNA, 45S rDNA, CAT36 (Kirov et al., 2017b), HAT58 (Kirov et al., 2017b) as well as AcepSAT750 and AcepSAT2500 (Peška et al., 2019) and the centromere repeat (this work) have been found. In addition, one cluster (CL137) identified by TAREAN software and corresponding to a 276 bp tandem repeat (TR2CL137) showed no similarity to the known *Allium* tandem repeats. Based on the read number the calculated genome portion and copy number of this repeat in *A. cepa* genome are 0.14% and 160,000 copies/2C, respectively. Comparison of the genome portion of the repeats between the two species showed clear differences for TR2CL137 and AcepSAT2500 being *A. cepa* specific and for HAT58 being *A. fistulosum* specific. CAT36, 5S rDNA, centromeric and subtelomeric repeats occupy a significantly higher portion in the *A. fistulosum* genome compared to the *A. cepa* genome (Figure 5A).

To study chromosomal organization of the newly defined tandem repeat TR2CL137 we performed FISH with this repeat on chromosomes of *A. cepa*. This demonstrated that TR2CL137 has a (peri)centromeric localization at chromosome 6 (Figures 5B,C). This chromosome already differs from other chromosomes in the *A. cepa* complement by the NOR region on the short arm. Thus, we identified here a novel chromosome-specific *A. cepa* repeat with (peri)centromeric localization on chromosome 6.

### Transcription of Centromeric DNA

**FIGURE 6:**
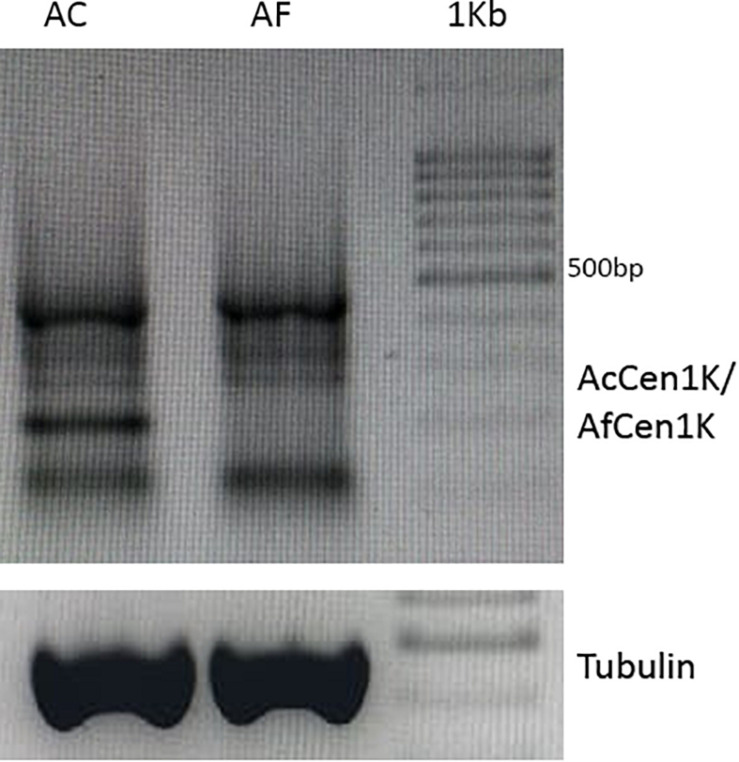
RT-PCR results with centromere specific primers and total RNA isolated from *A. cepa* and *A. fistulosum* seedlings. *Tubulin* was used as house-keeping reference gene (1 Kb–1 Kbp DNA Ladder (Syntol)).

Transcription of the CENH3-associated centromeric repeat was shown to be essential for centromere assembly (Topp et al., 2004; Talbert and Henikoff, 2018; Ideue and Tani, 2020). The previously published *A. cepa* and *A. fistulosum* RNAseq data (Tsukazaki et al., 2015) were used to assess the transcription of AfCen1K and AcCen1K tandem repeats. Mapping of RNAseq reads to the database containing reference transcriptomes and the centromere repeat as two tandemly organized units revealed no mapped reads for *A. fistulosum* and <10 mapped reads for *A. cepa* (RPKM, read per kilobase per million reads; value is <0.04), while the RPKM value for the actin reference transcript was 248 ± 78. Because the genomes of *A. cepa* and *A. fistulosum* possess a number of centromere repeat copies, the transcribed units can be diverged from the reference sequences of AcCen1K and AfCen1K. Therefore, we applied another strategy and performed mapping by BLAST to allow a lower divergence cutoff between the reads and the reference sequences. However, this did not significantly improve the results. We experimentally verified the results using RT-PCR with cDNA synthesized from the poly-A+ RNA fraction isolated from *A. cepa* and *A. fistulosum* seedlings and a centromere specific primer. Tubulin was used as a reference gene. No RT-PCR products were obtained with the centromere primers for both species although clear products were observed with the primers on tubulin gene. We next tested the presence of centromere transcripts in the non-polyA RNA fraction. For this, RT-PCR was carried out with total RNA (poly-A + non-poly-A RNAs) of *A. cepa* and *A. fistulosum*. This experiment revealed several RT-PCR products for *A. cepa* and *A. fistulosum* (Figure 6). We also predicted TSS and TATA-box motifs in the *Allium* CTRs using the TSSPlant (Shahmuradov et al., 2017) and plantCARE (for TATA-box only, Lescot et al., 2002) software. The results showed multiple standalone TATA-box sequences and one combination of the predicted TATA-box and TSS sites at position 1,159 bp (AfCen1K, TATA-box score = 8.1926; TSS score > 1.9) and 1,011 bp (AcCen1K, TATA-box score = 4.2; TSS score > 1.9). Thus, based on these results we concluded that AfCen1K and AcCen1K repeats are transcribed in *A. fistulosum* and *A. cepa* but the transcripts are not polyadenylated.

## Discussion

Here we showed that functional *A. fistulosum* centromeres contain tandemly organized and CENH3-associated repeat AfCen1K with monomer length ∼1.2 Kb. Moreover, we found that the genome of closely related species, *A. cepa*, possess similar repeat, AcCen1K, which differs from AfCen1K by an 632 bp long InDel sequence. Interestingly, FISH with AcCen1K and *A. cepa* chromosomes revealed no signals. These results together with our bioinformatics analysis of AcCen1K suggest that: (1) AcCen1K does not form long tandem arrays which is supported by the linear shape of RepeatExplorer cluster (Supplementary Figure 1), almost absence of discordantly mapped reads (Figure 3B) and absence of FISH signals on *A. cepa* chromosomes and (2) based on the detection of the AfCen1K-FISH signals on four *A. cepa* chromosomes (Figure 2A) it can be speculated that true high-copy *A. cepa* centromeric has similarity to AfCen1K-specific region. Our results are in line with reports from other plant species demonstrating a high divergence of CTR sequences between closely related species or even between chromosomes of one species (Jiang et al., 2003; Gong et al., 2012; Melters et al., 2013; Comai et al., 2017; Su et al., 2019; Robledillo et al., 2020; Talbert and Henikoff, 2020). To the best of our knowledge the monomer size of AfCen1K is one of the largest among CENH3 associated CTR known to date. The monomer size for most known CTRs shows a highly phased distribution with a peak around 150–180 bp corresponding to one wrap of the nucleosome (Melters et al., 2013; Yang et al., 2018). However, large CTRs monomers were previously characterized in Solanaceae (Gong et al., 2012), maize (Sharma et al., 2013), and Fabeae (Robledillo et al., 2018; Robledillo et al., 2020) species. Recent large-scale analysis of CENH3-associated CTRs in *Fabeae* indicated that 9 of 15 studied species have at least one CTR with monomer size >1,000 bp (Robledillo et al., 2020). This, together with our isolated long *A. fistulosum* CTR suggest that, in addition to sequence divergence, the plant centromeres exhibit broad diversity in CTR monomer length, varying over 170-fold: from 30 bp in *Vicia peregrine* (Robledillo et al., 2020) to 5,390 bp in *Solanum tuberosum* (Gong et al., 2012).

Using a paired-read approach, we found that *A. fistulosum* centromeres possess insertions of retrotransposons. Previously, we also found multiple copies of CR retrotransposon insertions into the centromeric region of *A. cepa* and *A. fistulosum* chromosomes (Kiseleva et al., 2014). In contrast, our bioinformatics analysis found Tekay and Retand Ty3/Gypsy retrotransposons to be more often associated with *Allium* centromeric repeats. But this data may also reflect that these retrotransposons have a high copy number in the *A. cepa* and *A. fistulosum* genomes. Indeed, Tekay retrotransposons are 37 times more abundant in *A. cepa* compared to the CR lineage (Peška et al., 2019). Origin of CTRs of some *Solanum* and *Vicia* species was associated with amplification of retrotransposons (Macas et al., 2009; Gong et al., 2012; Vondrak et al., 2020). Moreover, the CTRs exhibit detectable similarity to the LTRs or even to entire sequences of the putative parental retrotransposons. For example, the longest plant CTR, St3-294, identified in potato has high homology with the entire retrotransposon (Gong et al., 2012). Unfortunately, no *Allium* species have a reference library of mobile elements to which AcCen1K and AfCen1K can be compared to. Although we did not detect any similarity of the identified *Allium* CTRs to retrotransposon proteins, they can still be similar to the LTRs of retrotransposons. The LTR sequences of retrotransposons are highly variable between species and *Allium* LTRs are not present in the databases. Therefore, further annotation of full-length *Allium* retrotransposons should provide additional insight into their role in CTR evolution. We also found that AcCen1K and AfCen1K repeats are associated with insertions of chloroplast DNA. Moreover, we showed that centromeric region of some *A. cepa* and *A. fistulosum* chromosomes have insertions of chloroplast DNA. Chloroplast DNA integration into nuclear DNA is relatively frequently observed in plants (Huang et al., 2003). Moreover, the size of the chloroplast insertions vary from tens of base pairs to >100 Kb (Timmis et al., 2004; Huang et al., 2005; Noutsos et al., 2005; Sousa et al., 2016; Li et al., 2019). Our FISH experiments suggested that many of the insertions in *A. cepa* and *A. fistulosum* genomes have a length longer than 10Kb, the shortest chromosomal DNA target that can be routinely visualized by conventional FISH with mitotic chromosomes (Khrustaleva and Kik, 2001). While FISH signals were scattered along the chromosomes, few strong signals were observed in the primary constrictions (Figures 4C,D). These results are also supported by paired-end read analysis. In spite of low-coverage, NGS data were used and able to find paired reads with one mate with similarity to CTRs and the other mate with similarity to chloroplast DNA. This demonstrated that chloroplast insertions are located in close vicinity to AcCen1K and AfCen1K repeats. However, whether the chloroplast insertions are associated with CENH3 is unknown. Because of a low recombination frequency, the centromere region is a very favorable location for organelle DNA transfer suggesting their potential role in centromere evolution. It is therefore intriguing to check whether chloroplast DNA insertions may be coopted into CENH3-associated chromatin function and *de novo* CTR formation.

Transcription of CENH3 associated chromatin including centromeric retrotransposons and tandem repeats is now recognized as an important feature for centromere initiation and kinetochore assembly (Topp et al., 2004; Talbert and Henikoff, 2018; Liu et al., 2020; Talbert and Henikoff, 2020). Transcription of CTRs have been observed in a number of plant species including *Cucumis melo* (Setiawan et al., 2020) and *Arabidopsis*. We performed bioinformatic and RT-PCR analysis of AcCen1K and AfCen1K expression to detect RNA transcripts from these repeats. We found evidence of expression of these repeats in total RNA but not in the poly-A+ RNA fraction of seedlings. This suggested that polyadenylation of AcCen1K and AfCen1K transcripts is rare. The results are inconsistent with previous reports where polyadenylation was detected for CTR as well as CR transcripts (Topp et al., 2004; Lee et al., 2006). Interestingly, we predicted TSS and TATA-box sequences in AcCen1K and AfCen1K implying that they may serve as promoter triggering centromere transcription. Because the transcription of CENH3-associated DNA is essential for centromere assembly, evolution towards centromere repeat divergence may be constrained by a minimum sequence context required to trigger the transcription.

## Conclusion

Here, CENH3-associated functional CTR of *A. fistulosum* (AfCen1K, 1,239–1,259 bp) and its homologous sequence from *A. cepa* genome (AcCen1K, assumed monomer length – 1,255 bp) have been found. AfCen1K is located in all chromosomes of *A. fistulosum* while AcCen1K does not form long arrays in *A. cepa* genome and its location cannot be determined by FISH. An InDel polymorphism (632 bp) was detected between AcCen1K and AfCen1K sequences. AfCen1K and AcCen1K repeats are transcribed in *A. fistulosum* and *A. cepa* but the transcripts are not polyadenylated. Chloroplast DNA and mobile element insertions were found to be associated with AcCen1K and AfCen1K sequences. Moreover, large (>10 Kb) chloroplast DNA insertions were identified in the centromeric region of *A. fistulosum* and *A. cepa*. Finally, we identified a new species- and chromosome-specific tandem repeat of *A. cepa* located in the centromere region that can be used for further testing of its association with CENH3.

## Data Availability Statement

The raw sequencing data were uploaded to the NCBI (https://www.ncbi.nlm.nih.gov/sra/, SRA accession: PRJNA649851).

## Author Contributions

IK designed the present study. IK, SO, MO, PM, SG, MD, and AE performed the experiments. IK, MO, and LK analyzed the data. IK and MO performed the bioinformatics analysis. IK, KVL, LK, and AS participated in preparing and writing the manuscript. All authors contributed to revising the manuscript. All authors have read and approved the final manuscript.

## Conflict of Interest

The authors declare that the research was conducted in the absence of any commercial or financial relationships that could be construed as a potential conflict of interest.

## Footnotes

1 http://gydb.org

2 http://www.bioinformatics.nl/cgi-bin/primer3plus/primer3plus.cgi/

3 https://github.com/Kirovez/golang_progs/blob/master/random_pair.go

4 http://www.rstudio.com/

5 https://github.com/Kirovez/RepeatExplorer_scripts/blob/master/selectReadsUnpaired.py

6 http://drawid.xyz/
