# Supplementary material for: Functional Allium fistulosum Centromeres Comprise Arrays of a Long Satellite Repeat, Insertions of Retrotransposons and Chloroplast DNA
Source: Front Plant Sci. 2020 Oct 23;11:562001. doi: 10.3389/fpls.2020.562001 (PMC7644871; doi:10.3389/fpls.2020.562001)
Supplement: Supplementary file 1 [file Data_Sheet_1.docx]

**Supplementary Figure 1**. Read clusters build by RepeatExplorer2 and corresponding to *Allium cepa* (A) and *A. fistulosum* (B)


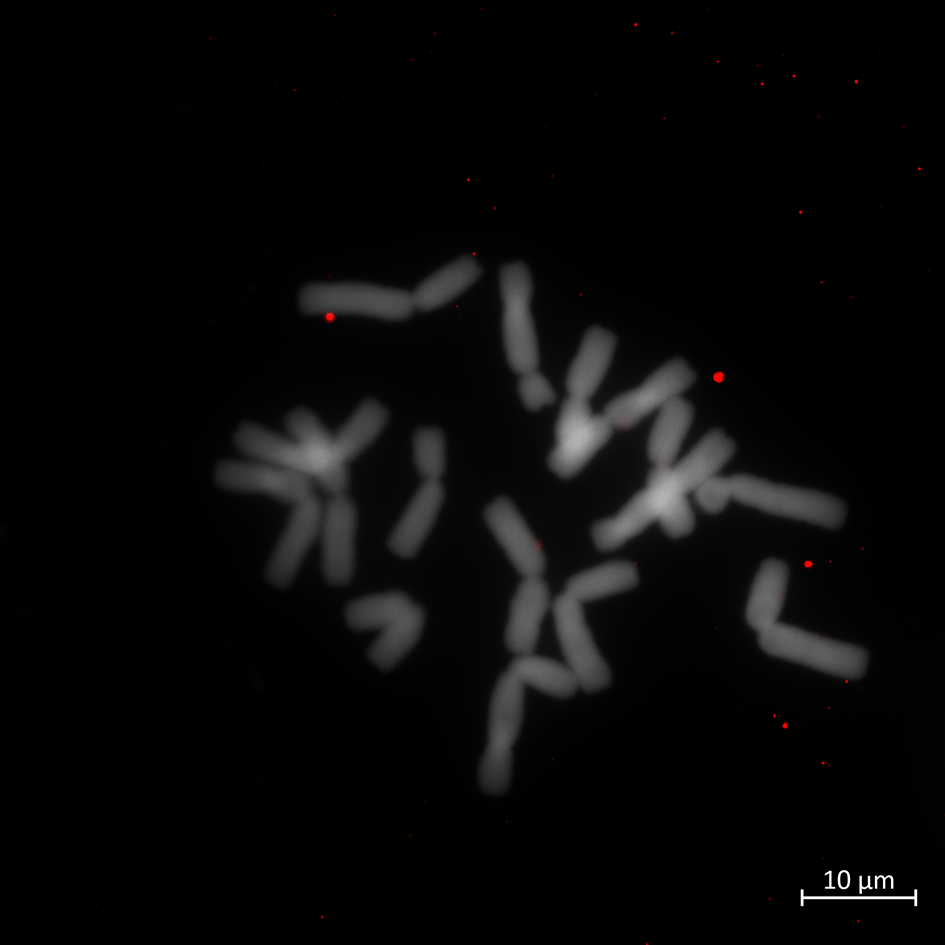


**Supplementary Figure 2**. FISH of labeled AcCen1K repeat of *A.cepa* with *A.cepa* chromosomes. Bar – 10 µm.


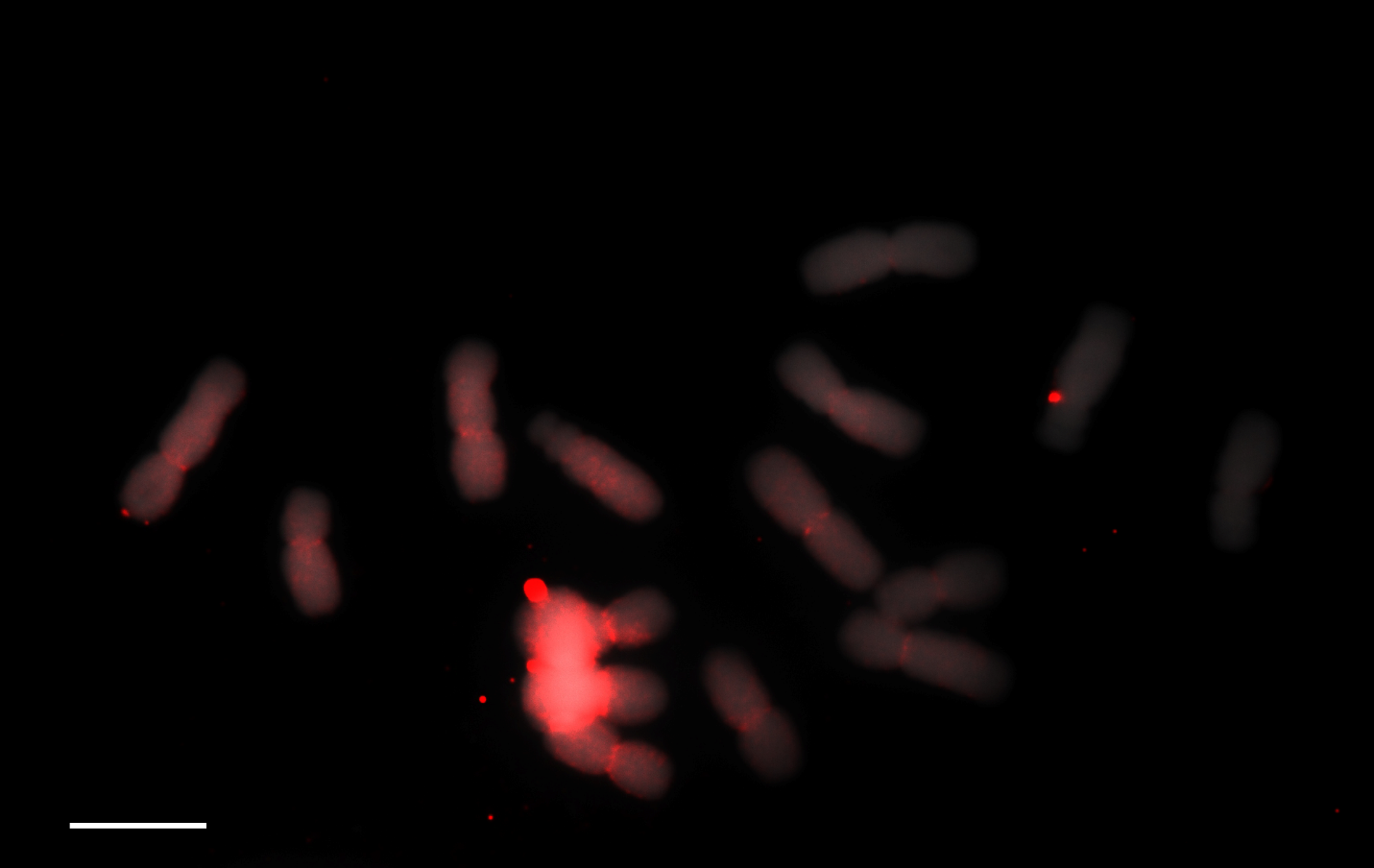


**Supplementary Figure 3**. FISH of labeled AcCen1K repeat of *A.cepa* with *A.fistulosum* chromosomes. Bar – 10 µm.


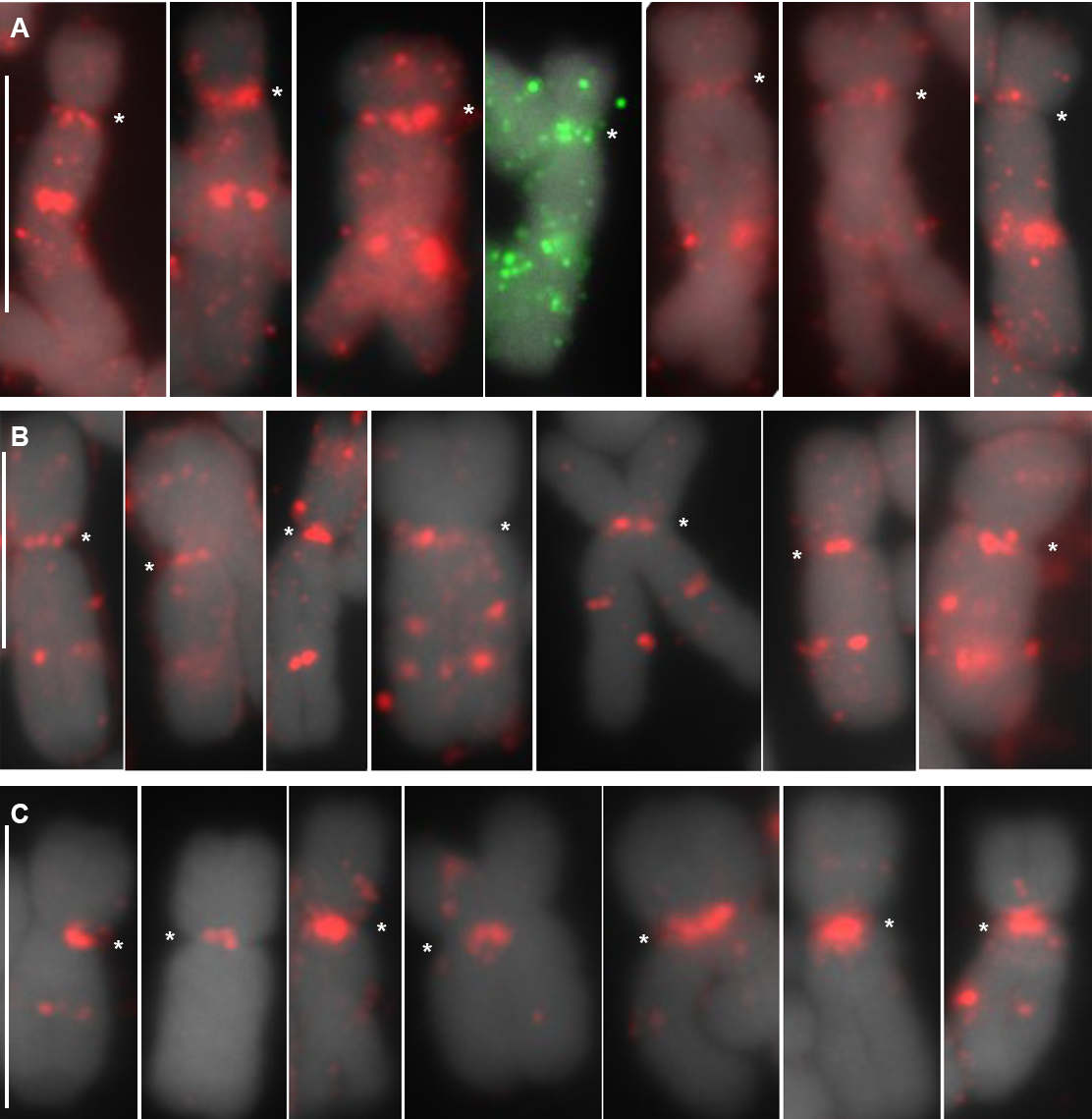


**Supplementary Figure 4.** FISH signals from hybridization of chloroplast DNA (A) *Allium cepa* chromosome 6 chromosome 6. (B) *A.fistulosum* chromosome 2. (C) *A.fistulosum* chromosome 8. Asterisks mark centromere position. Vertical white line indicates 10 µm.
