# Supplementary material for: Functional Allium fistulosum Centromeres Comprise Arrays of a Long Satellite Repeat, Insertions of Retrotransposons and Chloroplast DNA
Source: Front Plant Sci. 2020 Oct 23;11:562001. doi: 10.3389/fpls.2020.562001 (PMC7644871; doi:10.3389/fpls.2020.562001)
Supplement: Supplementary file 2 [file Data_Sheet_2.docx]

>AfCen1K_ref

TTAAATTTATAGATTCCGGCCTAAAATAGGTCCAT

GAACCTGTAAAAAATACTCGACGACCAGTACGGTGTACTAGCCCGAGACAATTCTTGGGT

TACTCCAGGATGTACAAATTTCTATAATATTGTATTTTTATTATTTTTAACTCTTTGTTC

TTGTGATTTCTTATCTTTTGATCTTCGTACTTACGTAATATACATTCATGATAAACCCTA

CTATTTTCCCTGTAACTTTTTACCCTCGTTTTATTTTTTAAGTTTATAATTCCTTCAATT

TCTTATATTGAAATAAATTATATAAATTCAATATATATAGCTTATATTCTAAATAAAATT

ATATTTCTTACTAAAACATATGTATTCGATCTATAGAAATCAAATCTTTATTTTTAAACC

GTATTATATATATATATATATATATCTATCAAATATTCCTGTATGTTATCACCGTTTAGT

GTCCTTTCACTTTTGAAATCAATTTCGTGCCTTTTTGGTGTGATTATCTACACCAAACCA

TTAATATACGAATTTGGATTGATTTTCATATAATATAATGTTTGTGTATATAAATTCATG

TAAATTTAATAGAATTGCATAAAAATACTAATTTTGGATATTATGGTTAATGAGAGCATCATGGAAGAAGAAGA

GGGAGGTGGAAAATTAGTTTTCTGAAATGGAAAGTGGGATACGCATCTCCTTTTTGAGTG

AAAGTAGATAAACAACTGCAATCTGCCCGAAAATTAAAATTTAGATTCAGATTGCGTGTC

GTCGGAGCGACTCGACGACGTTTTCGGTCTAATCTCCTTCATACGAGCTCCAAGCGACAC

AAACAAATATGTCGTTCAAATCAGAAAAACAAGACGGTTGCGATGGCTCCAGTTTCGTCA

CCAAATTCCAATGTCTTAACAATGACCATTTTACCCATCCTTCTTTTCAATTTATTTAAT

CAATCCATCGATTCCACTATTTTTCATACTATCATATTATTATTATTCAATTAGAATACT

ATTTGATTTTTCAAGGTATTAGATTCTCCCCATTAAAATGAATTTCGTCCCCAAATTCAA

AAACTAAGCCGGCTCTGTACCAAATCGTAAACCAAAATTAAGCCTGCTGTAACTTATAAA

TATTGCCGTTTTACGGCATGCGATACCCGTGTACTTAACGCTCATTGCCGCATAGGCTCG

GCGCCTATTCTACCACACATCACTCGGTCCAAGCACACTCAGGAAAGCAGGCATGAACCT

>AfCen1K_21

AATTTATAGATTCCGGCCTAAAATAGGTCCATGAACCTGTAAAAAATACTCGACGACCAGTACGGTGTACTAGCCCGAGACAATTCTTGGGTTACTACAGGATGTACAAATTTCTATAATATTGTATTTTTATTATTTTTAACTCTTTGTTCTTGTGATTTCTTATCTTTTGATCTTCGTACTTACGTAATATACATTCATGATAAACCCTACTATTTTCCCCGTAAGTTTTTACCCTCGTTTTATTTTTTAAGATTATATTCCTTCAACTTTCTTATATTGAAATAAATTATATAAATTCAATATATATAGCTTATATTCTAAATAAAATTATATTTCTTACTAAAACATATGTATTCAATCTATAGAAATCAAATCTTTATTTCTAAACCGTATTATATATATATATATCTATCTATCAAATATTCCTGTATGTTATCACCGTTTAGTGTCCTTTCACTTTTGAAATCAATTTCGTGCCTTTTTGATGTGATTATCTACACCAAACCATTAATATACGAATTTGGATCGATTTTCATATAATATAATGTTTGTGTATATAAATTCATGTAAATTTAATAGAATTGCATAAAATACTAATTTTGGATATTATGGCTAATGAGAGCATCATGGAAGAAGAAGAGGGAGATGGAAAATTAGTTTTCTGAAATGGAAAGTGGGATACGCATCTCCTTTTTGAGTCAAAGTAGATAAACAACTGCAATCTGCCCGAAAATTAAAATTTAGATTCAGATTGCGTGTCGTCGGAGCGACTCGACGACGTTTTCGGTCTAATCTCCTTCATACGAGCTCCAAGCGACACAAACAAATATGTCGTTCAAATCAGAAAAACAAGACGGTTGCGATGGCTCCAGTTTCGTCACCAAATTCCAATGTCTTAACAATGACCATTTTACCCATCCTTCTTTTTAATTTATTTAATCAATCCATCGATTCCACTATTTTTCATACTATCATATTATTTTTATTCAATTAGAATACTATTTGATTTTTCAAGGTATTAGATTCTCCCCATTAAAATGAATTTCGTCCCCAAATTCAAAAACTAAGCCGGCTCTGTACCAAATCGTAAACCAAAATTAAGCCTGCTGTAACTTATAAATATTGCCGTTTTACGGCATGCGATACCTGTGTACTAATACACATTGCGGCATAGGCTCTTTGCTTAATCTACCACATCACTCGGTCCGAGCACACTCCGGAAAGCAGGCATGAACCTTT

>AfCen1K_27

AAATTTATAGATTCCGGCCTAAAATAGGTCCAT

GAACCTGTAAAAAATACTCGACGACCAGTACGGTGTACTAGCCCGAGACAATTCTTGGGT

TACTCCAGGATGTACAAATTTCTATAATATTGTATTTTTATTATTTTTAACTCTTTGTTC

TTGTGATTTCTTATCTTTTGATCTTCGTACTTACGTAATATACATTCATGATAAACCCTA

CTATTTTCCCTGTAACTTTTTACCCTCGTTTTATTTTTTAAGTTTATAATTCCTTCAATT

TCTTATATTGAAATAAATTATATAAATTCAATATATATAGCTTATATTCTAAATAAAATT

ATATTTCTTACTAAAACATATGTATTCGATCTATAGAAATCAAATCTTTATTTTTAAACC

GTATTATATATATATATATATATATCTATCAAATATTCCTGTATGTTATCACCGTTTAGT

GTCCTTTCACTTTTGAAATCAATTTCGTGCCTTTTTGGTGTGATTATCTACACCAAACCA

TTAATATACGAATTTGGATTGATTTTCATATAATATAATGTTTGTGTATATAAATTCATG

TAAATTTAATAGA A

TTGCATAAAAATACTAATTTTGGATATTATGGTTAATGAGAGCATCATGGAAGAAGAAGA

GGGAGGTGGAAAATTAGTTTTCTGAAATGGAAAGTGGGATACGCATCTCCTTTTTGAGTG

AAAGTAGATAAACAACTGCAATCTGCCCGAAAATTAAAATTTAGATTCAGATTGCGTGTC

GTCGGAGCGACTCGACGACGTTTTCGGTCTAATCTCCTTCATACGAGCTCCAAGCGACAC

AAACAAATATGTCGTTCAAATCAGAAAAACAAGACGGTTGCGATGGCTCCAGTTTCGTCA

CCAAATTCCAATGTCTTAACAATGACCATTTTACCCATCCTTCTTTTCAATTTATTTAAT

CAATCCATCGATTCCACTATTTTTCATACTATCATATTATTATTATTCAATTAGAATACT

ATTTGATTTTTCAAGGTATTAGATTCTCCCCATTAAAATGAATTTCGTCCCCAAATTCAA

AAACTAAGCCGGCTCTGTACCAAATCGTAAACCAAAATTAAGCCTGCTGTAACTTATAAA

TATTGCCGTTTTACGGCATGCGATACCCGTGTACTTAACGCTCATTGCCGCATAGGCTCG

GCGCCTATTCTACCACACATCACTCGGTCCAAGCACACTCAGGAAAGCAGGCATGAACCT

>AfCen1K_45

AAATTTATAGATTCCGGCCTAAAATAGGTCCATGAACCTGTAAAAAATAC

TCGACGACCAGTACGGTGTACTAGCCCGAGACAATTCTTGGGTTACTCCAGGATGTACAA

ATTTCTATAATATTGTATTTTTATTATTTTTAACTCTTTGTTCTTGTGATTTCTTATCTT

TTGATCTTCGTACTTACGTAATATACATTCATGATAAACCCTACTATTTTCCCTGTAACT

TTTTACCCTCGTCTTATTTTTTAAGTTTATAATTCCTTCACTTTCTTATATTGAAATAAA

TTATATAAATTCAATATATATAGCTTATATTCTAAATAAAATTATATTTCTTACTAAAAC

ATATGTATTCGATCTATAGAAATCAAATCTTTGTTTTTAAACCGTATTATATATATATAT

ATCTATCAAATATTCCTGTATGTTATCACCGTTTAGTGTCCTTTCACTTTTGAAATCAAT

TTCGTGCCTTTTTGATGTGATTATCTACACCAAACCATTAATATACGAATTTGGATTGAT

TTTCATATAATATAATGTTTGTGTATATAAATTCATGTAAATTTAATAGAATTGCATAAA

AATACTAATTTTGGATATTATGGTTAATGAGA

GCATCATGGAAGAAGAAGAGGGAGATGGAAAATTAGTTTTCTGAAATGGAAAGTGGGATA

CGCATCTCCTTTTTGAGTCAAAGTAGATAAACAACTGCAATCTGCCCGAAAATTAAAATT

TAGATTCAGATTGCGTGTCGTCGGAGCGACTCGACGACGTTTTCGGTCTAATCTCCTTCA

TACGAGCTCCAAGCGACACAAACAAATATGTCGTTCAAATCAGAAAAACAAGACGGTTGC

GATGGCTCCAGTTTCGTCACCAAAWWMCAATGTCTTAACAATGACCATTTTACCCATCCT

TCTTTTTAATTTATTTAATCAATCCATCGATTCCACTATTTTTCATACTATCATATTATT

ATTATTCAATTAGAATACTATTTGATTTTTCAAGGTATTAGATTCTCCCCATTAAAATGA

ATTTCGTCCAAAATTCAAAAACTAAGCCGGCTCTGTACCAAATCGTAAACCAAAATTAAG

CCTGCTGTAGCATATAAATATTGCCGTTTTACGGCATGCGATACCTGTGTACTAATACAC

ATTGCGGCATAGGCTCTGTGCTTAATCTACCACATCACTCGGTCCGAGCACACTCCGGAA

ARCAGGCATGAACCTTT

>AfCen1K_12

AAATTTATAGATTCCGGCCTAAAATAGGTCCAGGAACCTGTAAAAAACACTCGACGACCAGTACGGTGTACTAGCCCGAGACAATTCTTGGGTTACTCCAGGATGTACAAATTTCTATAATATTGTATTTTTATTATTTTTAGCATTTTGTTTCTGGGATTTCTTAGCTTTTGATCTTCATACTTACATAATATACATTCATAATAAACCATACTATTTTTCCCGTAAGTTTTTACCCTCGTTTTGTTTTTTAAGATTATAATTCCTTCAACTTTCTTATATTGAAATAAATTGTATAAATTCAATATATATAGTTTATATTCTAAATAAAATTATATTTCTAACTAAAACATACTAAAACATACTAAAACATATGTATTCGATCTATAGAAATCAAATCTTTATTTTTAAACCGTATTATATATATATATAAATCATATATTCCTGTATGTTATCACCGTTTAGTGTACTATAACTTTTGAAATAAATTTCGTGCCATTTTGATGTGATTATCTGCACCAAACCATTAATATACGAATTTGGACTGCTTTTCATATAATATAATGTTTGTGCATATAAATTCGTGTAAATTTAATAGAATTGCATAAAAATACTAATTTTGGATATTATGGTTAATGAGAGCATCACGGAAGAAGAAGAGGGAGATGGGAAATTAGTTTTCTGAAATGAAAAGTGGGATACGCGTCTCCTTTTTGAGTCAAAGGAGATAAACAACTACAGTCTGCCCGAAAATTAAAATTCAGATTCAGATTGCGTGGCGTCGGTGCGACTCGACGACGTTTTCGGTCTAATCTCCTTCATACGAGCTCCAAGCGACACAAACAAATATATCGTTCAAATCAGAAAAACGAGACGGTTGTGATGGCGCCAGTTTCTTCACCAAATTCCAATGTCTTAAAAATGACCATTTTACCCATCCTTCTTTTTAATTTATTTAATCAATCCATCGATTCCACTATTTTTCATACTATCATATTATTATTATTCAATTAGAATACTATTTGATTTTTCAAGGTATTAGATTCTCCCCATTAAAATGAATTTCGTCCCAAAATTCAAAAACTAAGCCGGCTCTATACCAAATCGTAAACCAAAATTAAGCCTGATGTAACTTATAAATATTGCCGTTTTACGGCATGCGATACCCATGTACTAACACACATTGCCGCATAGGCTCGGTGCCTAATCTACCCCATCACTCGGTCCGAGCATACTCAGGAAAGCAGGCATGAACCTTT

>AcCen1K_assembled_from_NGS_reads

TGGAATCTAATGCCCCAAAAAATCAAATAGTATTCTAATTGAATATTGATAATATGACAA

TATGAAAAATAGTGGAATTGATGGATTGATTAACTAAATTAAAAAGAAGGATGGGTAAAA

TGGTCATTTTTAAGACTTTGGAATTTGGTGACGAAACTGGCGCCATCGCAACCGTCTCGT

TTTTCTGATTTGAACGATATATTTGTTTGTGTCGCTTGGAGCTCGTATGAAGGAGATTAG

ACCGAAAACGTCGTCGAGTCGCACCGACGCCACGCAATCTGAATCTGAATTTTAATTTTC

GGGCAGATTGCAGTTGTTTATCTCCTTTGACTCAAAAAGGAGATGCGTATCCCACTTTCC

ATTTCAGAAAACTAATTTCCCATATCCCTCTTCTTCTTCCCTGATGCTCTCATTAACCAT

AATATCCAAAATCAAAGCCAAATTAAGAAATTAAGGTCCCAAATCGAAGAGAGAAGCAAG

AAAGAATGCTTAGGGGTCACTGGTGGTGTTATAACAGCGAATCGAAGGAAGTGCTATCTC

TGCACAGCGAAAGAAAGTGAGGCTAGATGCTTTGGAATTGGGAGAAATCGATCATAGTAA

ATTCTATATTCACTCTCTTGATTTCATGATTTAATAAGACTATATTTGGTAACTAAATGT

GTAATTTCGAAATCAGCCCCTCTGAATTATGGAAACTTTGCCAAACATGAATAATGACTT

ATTTTTGAGTAGTTAAATGAGCTAATGGTGTAATATAATGTGCATCTAAACTCTATATGT

AAACTACGCGAAAATGAGGCATAAAATTTGGAAAATCATAATGAAATGAAGGATAGATCA

AGTTAAACTTATATGAGCAAATTTCTAAGATGAAATATGAGGATTACAGACCTAAACATA

ACCTTTGAGCATTCTTATACAATTTGTGCGAATTAATATGAGAATATTAAGTTTTTGTGC

AGAAACAGTCCAAAAATACAAATTTTATGATTCGGGCCATTTAAGTTTGTCCAATTGATA

TGAAACTGTCTTCTAAGATTTGATATGACGATATCAGGTCTAAGAAGTATTTTTATGCAA

TTCTATTAAATTTACACGAATTCATATACACAAACATTATATTATACGAAAAGCAGCCCA

AATTCGTATATTAATGGCTTGGTGTATATAATCACATCAAAATGGCACGAAATTGATTTC

AAAAGTTAAAGTACACTAAACGGTGATAACATACAAGAGTAAATGAGTTATATAT

>AcCen1K_clone1

ATTCTTGTATGTTATCACCGTTTAGTGTACTTTAACTTCTGAAATCAATTTCGTTCCATTTTGATGTGATTACACCAAACCATTGATATACGAATTTCGACTGCTTTTCATATAATACAACGTTCGTGCATATATATTCGTGTAAATTTAATTGAATTGCATAAAAATACTTTTTAGACCTGATATCATCATATCAAATCTCATAAGATAGCCTCAGACCAGTTGGATAAATTTAAATGGTCCGAATCATAAAATTTGAGATTTTTGGACTGTTTCTTCAGTAAAATTTATATTCTCACATTAATTCGCACAAATTGTATAAGAATGCTCAAAGATTATGTTTAGGTCCGTAGTCCTCATATTTCATCTTAGAAATTTGCTTATATAAGTTTAACTTGATCTATCCTTCATTTCATTATGATTATCCAAATTTTATGCCTCATTTTCTCGTAGTTTATATACAGAATTTAGATGTACATTGCATTAGACCATTAGCTCATTTAACTACTCACAAATAAATCATTATTCATGTTTGGCAAAGTTTCCAAAATTCAGAGGGGCTGGTTTTGAAATTATGCATTTAGTTACCAAATATAGTCTCATTAAATCATGAAATCAAGAGAGTGAATATAGAATTTACTACTATTGATTTCTCCCGATTCCAAAGCATAAAGCTACTTTCTTTCGCTGTGCAGAAAAAACACTTCCTACGATTCGCTGTTATAACACCACCCGTGACCCCTAAGCTTTCTTTCATGCCTCTCTCTTCGATTTGAGACCTTAATTTCTTAATTTGGCTTTGATTTTGGATATTATGGTTAATGAGAGCATCAGGGGAGAAGAGGAAGAAGAGGGAGGCGGGAAATTAGTTTTCTGAAACAGAAAGTGGATACGCATCTCCTTTTTGAGTCAAAGGAGATAAACAACCGCAATCTGTCCGAAAATTAAAATTCAGATTCAGATTGCGTGGCGTTGGTGCAACTCGACGACGTATTCGGTCCAATCTCCTTCATTCGAGCTCCAACCCACACAAACAAATATATCGTTCAAATCGGAAAAACGAGACGGTTGCGATGGCGCCAGTTTCGTCA

>AcCen1K_clone2

GACGAAACTGGCGCCATCGTAACCATCTCGTTTTTCTGATTTGAAAGATATATTTATTTATGTCGGTTGGAGTTCGTATGAAGGATAGTAGACCGAAAATGTCATTGAGTCGCACTAACGCCTCCCATTCTGAATCTGAATTTTAATTTTCGGGCAGATTGCAGTTGTTTATCTCCTTCGACTCAAAAACGCAATGTGTATCCCACTTTCCATTTCAGGTAACTAATTTTCCATCTCCCTCTTCTTCTTCCCTGATGCTCTCTTTAACCATAATATCCAAAGTAAAAGCCAAATTAAGGTCCCAAATCGAAGAGAGAAGCAAGAAAGAATGCTTACGGGTCGCTGGTGGCGTTATAACAGCGAATCGAAGTAAGTGCTATCACTGCACAGCAATAGAAAGTGAGGCTAGATGCTTTGGAATTGGGAGAAATCAATCATAGTAAATTCTATATTCACTCTCTTGATTTCGTGATTTAATAAGATTATATTAGGTAAGTAAATGTCTTATTTCGAAATCAGGCTGTCTGAACTTTGGAAACTTTGCCAAACATGAATAATGATTCATTTTGGAGTAGTTAAATGATCTAATGGTGTAATATAATGTTCATCTAAACTCTATGTTAACTATGCGAAAATGAGGCATAATATTTGGAAAATCATAATGAAATCAAAGAACGGATCAAGTTAAACTTATGAGAGCAAATTTCTATGATGAAATACGATGATTACGGAATTAAACGTAATATTTGAGCATTCTTATACAGTTTGTGTCAATTAAAATGAGAATGTTAAGTTTTTTGTGCAAAAAACAGTCCAAAAAATATAAATTTTTATGATTTCGGGGCCATTTTAAGTATGTCCAACTAATATGAAACTATCTTCCGAGATTTGATATGATGATGTCAGGACTAAAAAGTATTTTTATGTAATTCTATTAAATTTACACGAATTTATACACGCAAAAATTATATTATACGAAAAGCAGTCCAAATTCGTATATTAATGGTTTGGTGTACGTGATTACATCAAAAAGGCACGAAATTGATTTCAAATGTTAAGGTACACTAAACGGTGATAACATACAAGAA

>AcCen1K_clone4

GACGAAACTGGCGCCATCGTAACCATCTCGTTTTTCTGATTTGAAAGATATATTTATTTATGTCGGTTGGAGTTCGTATGAAGGATAGTAGACCGAAAATGTCATTGAGTCGCACTAACGCCTCCCATTCTGAATCTGAATTTTAATTTTCGGGCAGATTGCAGTTGTTTATCTCCTTCGACTCAAAAACGCAATGTGTATCCCACTTTCCATTTCAGGTAACTAATTTTCCATCTCCCTCTTCTTCTTCCCTGATGCTCTCTTTAACCATAATATCCAAAGTAAAAGCCAAATTAAGGTCCCAAATCGAAGAGAGAAGCAAGAAAGAATGCTTACGGGTCGCTGGTGGCGTTATAACAGCGAATCGAAGTAAGTGCTATCACTGCACAGCAATAGAAAGTGAGGCTAGATGCTTTGGAATTGGGAGAAATCAATCATAGTAAATTCTATATTCACTCTCTTGATTTCGTGATTTAATAAGATTATATTAGGTAAGTAAATGTCTTATTTCGAAATCAGGCTGTCTGAACTTTGGAAACTTTGCCAAACATGAATAATGATTCATTTTGGAGTAGTTAAATGATCTAATGGTGTAATATAATGTTCATCTAAACTCTATGTTAACTATGCGAAAATGAGGCATAATATTTGGAAAATCATAATGAAATCAAAGAACGGATCAAGTTAAACTTATGAGAGCAAATTTCTATGATGAAATACGATGATTACGGAATTAAACGTAATATTTGAGCATTCTTATACAGTTTGTGTCAATTAAAATGAGAATGTTAAGTTTTTTGTGCAAAAAACAGTCCAAAAAATATAAATTTTTATGATTTCGGGGCCATTTTAAGTATGTCCAACTAATATGAAACTATCTTCCGAGATTTGATATGATGATGTCAGGACTAAAAAGTATTTTTATGTAATTCTATTAAATTTACACGAATTTATACACGCAAAAATTATATTATACGAAAAGCAGTCCAAATTCGTATATTAATGGTTTGGTGTACGTGATTACATCAAAAAGGCACGAAATTGATTTCAAATGTTAAGGTACACTAAACGGTGATAACATACAAGAA

>AcCen1K_clone5

GACGAAACTGGCGCCATCGCAACCATCTCGTTTTTCTGATATCGATATATTTGTTTCGTCGCTTGGAGCTCGTATGAAGGAGATTAGACAGAAAACGTCGTCGAGTCGCACTGACACCACGCAATCTGAATCCGGATTTTAATTTTCGGGCAGATCGCAGTTGTTTATCTCCTTTGACTCAAAAAGGGGATATGTGCCCCATTTTCCATTTCAGAAAACTAATTTTCCATATTTCTCTACTTTAACCGTAATATCCAAAATCAAAGCAAAATTAAGAAACTAATCTCCCAAATCGAGCAGAGAAGCAAGTAAGAATGAATGCTTAGGGGTCCACTGATGGTGTTATAACAGCAAACTGAAGGAGTACTATCTCTGCACAGCGAACGGAAGTGAGGCTAGAGGCTTTGGAATTGGGAGAAATCGATCATAGTAAATTATTCACTCTCTTGATTTCATGATTTAATAATATTTGGTAGTGAAACGTGTAATTTCAAAATTAGGCTCTCTGAATTGTGGAAACTTTGCCAAACATGAATAATGACTTATTTTTGTATAGTTAAATGGGCTGATGGTGTATATGTAAACTACGCGAAAATGAGGCATAAAATTTGGAAAATCATAGTGAAATGAAGGATAGATCAAGTTAAACTTATATGAGCAAATTGCTAAGACGAAATATGATGTTTAAAGAGGCCTAAACATAATTTCGAGCACTCTTACTCAATTTGTTTGAATTAATATGATAATATTATGTTTCTGTCCAGAAACGGTCCAAAAAATACAAATTTTATAATTCGGGCCCCTTTATGTTTGTCCAATTGATATGAACCTATCTTCTGAGATTCGATACGATGATATCGGTTCGAAAAAGTATTACCGTGGAATTATATTTAAATTTACATGAATTCGTATACAAAAACATTATATTATATGAAAAGCAGTCCAAATTCGTATATCGATGGTTTGGTGTATATAATCACATCTAATGGCAAGAAATTGATGTCAAAAGTTAAAGTACACTAAACGGTGATAACATACAAA

>AcCen1K_clone7

TCTTGTATGTTATCACCGTTTAGTGTACTTTAACTATTGAAATCAATTTCGTGCCAAGTTGATGTGATTATACACACCAAACCATCAATATACGAATTTGGACTGCTTTTCATATGATATAATGTTTCTGTATAAGAATTCGCATAAATTTCAATAAAATTACTTAATAATACTTTTTAGACCTCATATAATCGTATCAAATCTCAGTAGATAGATCCATGTCAATTGGACGAACTTAAAATGCCCGAATCATAAAATTTGTATTTTTGGTCCGTTTCTGCACAAAAACATAATATTCTCATATTTATTCGAGCAAATTGTGTATGAGTGCTTGAACATTATGTTTAGGTCACTGTAAACGTCATATTTCATCTAAGTAATTTGCTCATATAAACTTGATATATCCTTCATTTCACTATTATTTTCCAAATTTTTTGCCTCATTTTCGCGTAGTTTACATACAGAGTTTAGATGCACATTATGTTACACCATTAGCCCATTTAACTATACAAAAATAAGTCATTATTCACGTTTGGCAAAGTTTGCATAATTCAGAGAGCCTATTTCGAAATTACACATTTAGTTACCAAATATTATTAAATCATGAAATCAAGAGAGTGAATATAGAATTTATTATGATCGATTTCACCCAATTCCAAAGCTACTAGCCTCACTTCCTTTCGATGTGCAGAGATAGCACTCCTTAAGTTCGCTGTTATAACACCACCAGTGACCCCTAAGCTTTCTTTCTTGCTTCTCTCTTCGATTTGGGACCTTCATTTCTTAATTTTGCTCTGATTTTGGATATTATGTTTAATGAGAGAGTAAGGGGAGAAGAAGAGACATATGAGAAATTAGTTTTCTGAAATGGAAAGTGGGATAGTATCACCTTTGGGAGTCAAAGGAAATAAGCAACTACAATCTGACCGGAAATTAAAATTCGGATTCAGATCGCGTGGCGTCGGTGCGACTTGACGACGTTTTCGGTCTAATCTCCGTCATACGAGCTCCAAGCGGCGCAAACAAATATATCGTTCAGATCAAAAAAACGAGACGGTTGCGATGGCGCCAGTTTCGTCA

>AcCen1K_clone10

GACGAAACTGGCGCCATCGCAACCGTCTCGCTTTTCTGATTTGAACGATATATTTGTTAGTGTCGCTTAAAGCTCGTATGAAGGAGATTAGACCGAAAACGTCGTCGAGTCGCACCGAAGCCACGCAATCTGAGTCCGAAGTTTAATTTTCGGTCATATTGCAGTTGTTTATCTCCTTTGACTCCACAAGGAGATACGTATCCCACTTTCCATTTCAGAAAACTAATCTCCCATATGTGTCTTCTTCTTCCCTAACGCTCTCATTAACCATAATATCCAAAATCAAAGCAAAATTAAGAAATTAAGGTCCCAAATCGAAGAGAGAAGCAAGAAAGAATGCTTAGGGTCACCGGTGGTGTTACAACAGCAAATTGAAGGAGTGCTATCTCTGCTCTGCGAAAGGAAGTCAGGGTAGAGGCTTTGGAATTGGGGTAAAATCTATCATAGTAAATTCTATATTCACTCTCTTGATTTCATGATTTAATAATATTCGGTAACTAAATGTGTAATTTTGAAATTAGGCTCTCTGAGTTCTGGAAACTTTACCAAACATGAATAATGACTAATTTTTGTGTAGTTAGATGGAGCTAATGGTGTAATAAAATGTGCATCTAAACTCTATATTTAAACTACGCAAAAATGAGGCATAAAATTTGGAAAATCATAGTGAAGTGAAGGATAGATCAAGTTAAACATATACGAGCAAATTGCTAAGATGAAATATGACGACTACAGACACCTATTCATAATCTCTGAGCACACTTACACAATTTGTTCGAATTAATATGATAATATAATGTTTCTTTGCAAAACGGTCCAAAAAATACCAAATTTACTATTCGGGCCCTTTACGTTTGTCCATTTGATATGAACCGATCTTCTGAGATTTGATACGATGATATCAGGTCGAAAAAGTATTACTGTGGAATTCTATTGAAATTTGCACAACTCGTATACAAAAACGTTATATTATATGAAAAGCAGTCCAAATTCGTATAATGTTGGTTTGGTATATATAATCTCATCAAAATAGCACGAAATTGATTTCAAAAGTTAAAGTACACTAAACGGTGATAACATACAAGAA
